# Oil spill response capabilities and technologies for ice-covered Arctic marine waters: A review of recent developments and established practices

**DOI:** 10.1007/s13280-017-0958-y

**Published:** 2017-10-28

**Authors:** Jeremy Wilkinson, CJ Beegle-Krause, Karl-Ulrich Evers, Nick Hughes, Alun Lewis, Mark Reed, Peter Wadhams

**Affiliations:** 1 British Antarctic Survey, High Cross, Madingley Road, Cambridge, CB3 0ET UK; 2 SINTEF Ocean AS, Postboks Box 4762, Torgard, 7465 Trondheim, Norway; 3 Hamburgische Schiffbau-Versuchsanstalt GmbH, Hamburg Ship Model Basin (HSVA), Bramfelder Straße 164, 22305 Hamburg, Germany; 4 P.O. Box 6314, Kirkegårdsv. 60, 9293 Tromsø, Norway; 5 121 Laleham Road, Staines-upon-Thames, Middlesex, TW18 2EG UK; 6 Høgåsen 6, 7560 Vikhammer, Norway; 7 DAMTP, Centre for Mathematical Sciences, Wilberforce Road, Cambridge, CB3 0WA UK

**Keywords:** Arctic, Oil spill response, Sea ice

## Abstract

Renewed political and commercial interest in the resources of the Arctic, the reduction in the extent and thickness of sea ice, and the recent failings that led to the Deepwater Horizon oil spill, have prompted industry and its regulatory agencies, governments, local communities and NGOs to look at all aspects of Arctic oil spill countermeasures with fresh eyes. This paper provides an overview of present oil spill response capabilities and technologies for ice-covered waters, as well as under potential future conditions driven by a changing climate. Though not an exhaustive review, we provide the key research results for oil spill response from knowledge accumulated over many decades, including significant review papers that have been prepared as well as results from recent laboratory tests, field programmes and modelling work. The three main areas covered by the review are as follows: oil weathering and modelling; oil detection and monitoring; and oil spill response techniques.

## Introduction

Politics, economics and climate change are the driving forces behind the ‘industrialisation’ of the Arctic marine environment. Which of these three forces are more dominant is far from certain, but what is clear is that any increase in human activity in ice-covered waters will magnify the potential for an oil spill. Whether it be from a shipping accident, leak from a subsurface pipeline, subsurface well blowout or a cruise ship venturing further into shallow waters.

Whilst we have seen a substantial increase in Arctic fisheries and tourism, the recent slump in world oil prices combined with the need to reduce our carbon footprint in line with the legally binding Paris Climate Agreement has potentially reduced the attractiveness of investment in the Arctic. For instance, a number of major oil companies have announced the abandonment or suspension of their drilling operations in the Arctic Ocean, and trans-Arctic shipping remains at low levels. However, operations do continue, for example the newly built offshore terminals in Kara Sea region of the Russian Arctic handled (by ship) a combined 230 000 barrels a day in the second quarter of 2016 (Lee 2016). The potential for an Arctic sea route for Canadian oil-sand bitumen would create known and new oil spill response issues (Environment Canada 2013; NOAA 2013). Whilst the navigation of vessels through sea ice may be more challenging, we note that in 2016 more oil was shipped out of these Russian terminals during the sea ice season than during the previous open water season. This suggests that with the correct infrastructure and vessels, ice conditions may not be a limiting factor for the movement of oil (or indeed other valuable commodities) out of, through, or into the region. Then again, as this manuscript details, the presence of sea ice enhances the difficulty of clean-up operations should a major spill happen.

In common with oil spill response in temperate, open-ocean conditions, the purpose of conducting any oil spill response in ice-covered waters is to reduce the damage that the spilled oil might cause, both ecological and socio-economic. Knowledge of which socio-economic, ecological or cultural resources are likely to be damaged by the spilled oil at any particular location is important, so that the appropriate response strategies and methods can be used to minimise the damage that could occur. For example, cultural resource protection (e.g. remaining “secret”) requires knowledgeable representatives to participate in Shoreline Cleanup Assessment Technique (SCAT) and response operations to prevent direct oiling, accidental disturbance, illicit collecting or intentional site disturbance (Owens et al. 2005).

The degree of effectiveness of the response is the degree to which the damage by the oil is reduced when compared with a no response action. One of the challenges of accurately quantifying the damage of an Arctic oil spill is that our baseline knowledge of the Arctic system is presently limited. Over the years, there have been a number of reviews (some with recommendations) into Arctic oil spills; these include the following:

- *National Academy of Sciences’* report on responding to oil spills in the U.S. Arctic marine environment (NRC 2014);
- *JIP* The Joint Industry Programme’s series of advanced research projects and reports on: dispersants, environmental effects, trajectory modelling, remote sensing, mechanical recovery and in situ burning (JIP 2016);
- *USGS* The United States Geological Survey’s evaluation of the science needs for informed decisions on energy development in the Chukchi and Beaufort Seas (Holland-Bartels et al. 2011);
- *PEW Charitable Trust’s* review on Arctic Standards (Pew Charitable Trust 2013); and
- *Coastal Response Research Center’s* (University of New Hampshire) review of the state-of-science for dispersant use in Arctic waters (CRRC 2016).

An earlier, but still a comprehensive study, the Canadian Government Beaufort Sea Project (1974–1980) with 40 reports, focuses on different aspects of an oil spill in the Arctic marine environment. There have been notable experimental and unplanned oil releases in the Arctic: the Dome Petroleum Experiment (1979/1980) (Dickins et al. 1981), the Komi oil spill (1994) (Sagers 1994) and the Joint Industry Program Field Experiment (2009) (Sørstrøm et al. 2010). More recently, a review performed by the Royal Society of Canada (Lee et al. 2015) focuses on crude oil releases in freshwater and saltwater environments, but has information on the risks associated with Arctic oil spills. Much new research is coming out now through the Arctic Response Technology Joint Industry Program, which funded nine projects exploring the movement, fate, and effects of oil, detection of oil in ice, and oil recovery, in situ burning and potential use of chemical herders (JIP 2016).

This paper brings together knowledge that has been amassed over many decades, including the significant review papers mentioned above, as well as more recent laboratory tests, field programmes and modelling work. The three main areas covered by the review are as follows: (1) Weathering and modelling, (2) Oil detection and monitoring and (3) Oil Spill Response Techniques. We understand that there are omissions that given space restrictions we could not include. These include amongst others: biodegradation, effects of oil on Arctic ecosystems, infrastructure needs, logistics, training and education, indigenous communities perspectives and representation, chain of command/coordination, and the ethics, regulatory and international framework encompassing a potential Arctic oil spill.

### Oil and sea ice

Depending on the season, the sea ice conditions at the time of the event and type of accident (whether it be a pipeline breach, well blowout, shipping accident, or something else), oil could be spilled on, under, or into the waters surrounding the sea ice. However, what makes an Arctic oil spill particularly challenging is the plethora of environmental scenarios that could play out and the speed in which ice conditions can change. Furthermore, the combination of natural variability and climate-forced changes in the Arctic marine system make it particularly challenging to predict the ice conditions from one year to the next. Even though a spill could happen at any time of the year, it is important to keep in mind that most Arctic marine activities, at present, are concentrated around the summer months, and generally avoid sea ice. This summer focus may change as operational experience is gained; infrastructure is enhanced, and the continued increase in the ice-free season, over the next 30 years and more, stretches into other seasons.

#### Oil movement in sea ice

Oil spilled on a calm ocean surface spreads into a slick due to the balance between the forces of gravity, viscosity and surface tension. In rougher water, this spreading is augmented significantly by the entrainment of oil droplets into the water column by breaking waves, and subsequent resurfacing. The trajectory or drift of the slick is governed by the forces associated with currents, winds and waves (Wang et al. 2005). Sea ice adds a new dimension to the movement of oil, and therefore, understanding how far oil spilled on sea ice-infested waters will spread is of particular importance.

In summer, the sea ice zone is a particularly challenging environment because the concentration of ice floes within a region is continuously changing. Oil spilt in these conditions will generally gather on the surface among the floes, but wind and current can move the floes together squeezing the oil between them, or drift apart allowing the oil to spread out over a larger area of the sea surface. Venkatesh et al. (1990) suggested that for low sea ice concentrations (less than 30%) oil behaved as in open water, and for ice concentrations higher than 70–80%, they found that oil drifts with ice. The gap, between 30 and 70% ice concentration, is a transition zone which requires further research. Yapa and Weerasuriya (1997) developed a theoretical model for oil behaviour under drift ice by modifying earlier work on oil under ice to allow for oil escape through cracks.

In winter, oil present in these open water regions, known as leads, is likely to be incorporated in any newly formed ice. If the lead closes, oil incorporated within the new ice will form the blocks of the pressure ridge, essentially making the oil inaccessible for clean-up operations. However, if the oil is released below the ice cover, from a sunken vessel, pipeline breach or well blowout, the oil will rise through the water column breaking down into small droplets as it rises at the transition point of the multiphase plume driven flow (Johansen et al. 2013). In the case of a blowout, it is important to remember that oil and gas will be released together. The effect of the oil/gas mixture has on the sea ice is not fully established, but when the oil itself reaches the underside of the ice most of oil droplets will coalesce to form an oil slick. As the oil layer thickens, the slick will then move outwards from the central region due to hydrostatic pressure differences. Laboratory and in situ testing under a flat ice bottom suggest that the maximum thickness range for oil free to spread is 0.5–1 cm (Dickins et al. 1975; Keevil and Ramseier 1975), depending on the oil properties.

**Fig. 1:**
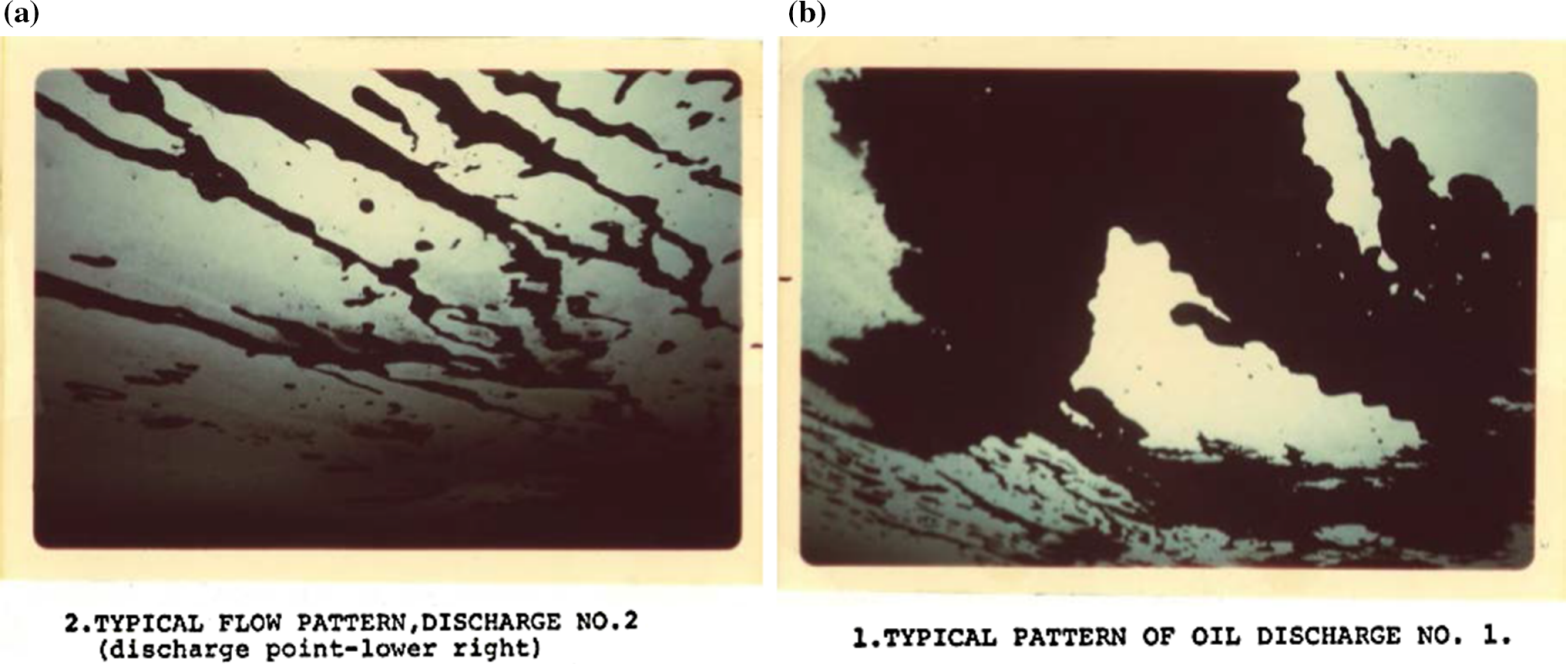
**a** Oil forming small rivulets that move from one depression to the next. NORCOR oil under ice recovery tests Beaufort Sea, May 1975 (also after Wadhams 1976a, b). **b** Oil gathers in depressions to form under ice larger rivers. Also visible in the image are areas of thicker ice remaining as ‘clean’ islands surrounded by oil. NORCOR (1975) (also after Wadhams 1976a, b)

The oil will then move outwards beyond the spill zone filling all available irregularities, but preferentially flowing towards regions of thinner ice. This movement will either be dominated by the oil spreading out in narrow rivulets (Fig. 1a) or filling up deeper and wider depressions such as those seen in Fig. 1b. When an individual depression is full, a rivulet of oil run will flow outward over the depression and into the next interconnected depression (Fingas and Hollebone 2003; Wilkinson et al. 2007).

Whilst under-ice roughness is a dominate factor that controls an oil’s movement there are other factors that influence the rate at which oil spreads under sea ice, including the rate at which the oil is introduced, the oil viscosity, and the surface oil–ice–water interfacial tensions (Wadhams 1976a, b, 1980; Malcolm 1979; Wilkinson et al. 2007). The direction of the flow of oil is a function of the under-ice topography, ice dynamics upper ocean turbulence and oceanic currents. Individual sessile drops or slicks located under the ice are quite difficult to move by ocean currents. This is due to the “sticking friction” between the drop and the skeletal layer at the ice/water interface (Lewis 1976). Tests to quantify the movement of oil due to oceanic currents have shown that the minimum threshold current to move crude oil under smooth sea ice was in the order of 0.15 m/s increasing to approximately 0.21 m/s under slightly rougher ice (Cox and Schultz 1980).

It should be noted that under drifting ice any rising oil will “paint” the underside of the ice irregularly, giving a large number of small under-ice slicks, while under fast ice a much larger oil pool may form because the ice is not in motion (Lewis 1976; Wadhams 1989, 2012).

#### Oil migration

If the density of oil is lower than the surrounding seawater then oil will attempt to migrate upwards into the sea ice. This upward migration is limited when the ice is cold. During a series of laboratory-grown sea ice experiments, Karlsson et al. (2011) found that the bottom skeletal layer of cold ice, immediately above the oil, was oil-saturated. They concluded that 5% of the bottom 2 cm is saturated with oil. Even within cold ice, they found oil can reach several centimetres into the ice through discrete brine channels. Interestingly, experiments performed in early 1970s by NORCOR Engineering and Research Limited, which were undertaken within an older and thicker ice regime, also found that the bottom few centimetres were heavily contaminated, with examples of discrete oil penetration (via brine channels) to around 5–10 cm above ice bottom (NORCOR 1975; Martin 1979).

Karlsson et al. (2011) quantified oil uptake capacity of these lower skeletal layers as being around 1 l/m^2^ during the sea ice growth season, whilst Petrich et al. (2013) provided significant higher estimates (based on the characteristics of landfast ice at Barrow, Alaska), of up to 10 l/m^2^. These higher estimates, when compared to holding capacity of oil under first year ice from Wilkinson et al. (2007), suggest entrainment may reach approximately 20% of the potential oil volume pooled beneath sea ice. However, Maus et al. (2013) pointed out that care must be taken when interpreting laboratory experiments, due to limited amounts of oil released, and possible differences in ice characteristics.

Under warmer conditions, e.g. spring–summer transition, encapsulated oil or oil located at the bottom of the ice can move vertically upwards through the ice, until it reaches the ice surface; a process known as oil migration. Field and laboratory studies indicate that oil under or encapsulated within sea ice will be released as the ice warms up, and this release will be either through vertical migration of oil, or through the ablation/melt of the ice surface downwards (e.g. Lewis 1976; Martin 1979; Dickins 2011). These methods transfer significant amounts of oil from within or under the ice to the ice surface or overlaying melt ponds.

The oil migration process is not well understood, both due to a lack in field observations, and due to incomplete knowledge of sea ice microstructure evolution during melt and growth (Maus et al. 2015). Field-based evidence from older ice, of both these processes at play, can be found in Martin (1979) and NORCOR (1975). During this study, Martin (1979) spilled oil under land-fast sea ice (of 1.5–2 m thickness) in February and April 1975. By the end of May melt pools had formed on the ice surface and oil had begun to migrate up to the surface. Whilst the NORCOR results do not mention accompanying ice porosity measurements, Karlsson et al. (2011) suggested that this migration is triggered when the warming ice reaches a certain porosity threshold, although Maus et al. (2015) did not find this porosity threshold link. As yet no models have been developed to parameterise oil releases events through ice ablation and oil migration (Fingas and Hollebone 2003). Understanding and predicting the timing of oil migration and surface release better is important for logistics of clean-up, and evaluation of areas affected during ice drift (Maus et al. 2013).

#### Oil encapsulation

If oil at the bottom of the ice is present during the ice growth season, then growing sea ice may form a lip around the perimeter of the oil pool under the ice, inhibiting the further horizontal spread of the oil. Results from Lewis (1976) and Izumiyama et al. (2002) suggest that ice growth under the oil layer is reduced due to the insulating properties of oil compared to ice. If the transfer of heat from the ocean/atmosphere to the ice–oil–ocean interfaces is sufficient for ice growth, the ice will continue to grow beneath the oil pool eventually completely encapsulating the oil within the ice matrix (NORCOR 1975) and forming what is known as an “ice-oil sandwich”.

In situ experiments in March (Beaufort Sea region) showed that an oil pool oil, spilled under 160-cm-thick ice, took about a week for ice to begin to encapsulate the oil (Lewis 1976). The encapsulation process is dependent on the ice growth process at the bottom of the ice, which in turn is controlled by the ocean–atmosphere heat flux, and as such is influenced by the thickness of the ice and snow above the oil. During the ice growth season, the encapsulation process would be quicker under thin ice, and may not happen at all under thicker ice. While the oil is encapsulated some water-soluble compounds in the oil may be dissolved with the brine and released into the ocean during ice growth (Faksness and Brandvik 2008).

## Weathering and modelling

Accidental oil spills in the marine environment, whilst rare, are not an unknown phenomenon, and as such the oil spill modelling community is well versed in the fate of oil in more temperate environments. Complex models have been developed that use oceanographic, atmospheric, and weathering data to determine both the trajectory and fate of an oil spill in the open ocean. It is generally accepted that these complex models are well established and do a reasonable job, however similar modelling scenarios in the presence of sea ice are much more uncertain (Johansen et al. 2005). This is understandable, as most of the ship traffic and hydrocarbon exploration and exploitation to date have occurred in the ‘warm’ seas, far away from floating ice. However, the Arctic is changing. For example, oil exportation and exploitation in the Arctic are ongoing; ‘fact-finding’ commercial shipping is already transiting the Northern Sea Route; tourist and fishing vessels are increasing in number, and ice-strengthened oil tankers are regularly plying the seasonally ice-locked oil terminals in the Russia sector of the Arctic. Consequently, accurate and reliable models on the movement and fate of oil in ice-covered seas are needed now more than ever. This is especially important when one considers the challenges climate change presently brings to the region, as well as those that are predicted in the future.

The main mechanisms which govern the fate or weathering of an oil slick are spreading, evaporation, dispersion, emulsification, sedimentation and biodegradation. It is widely recognised that oil weathering is strongly dependent on the specific chemical composition and characteristics of individual crude oils (Daling and Strom 1999; Brandvik et al. 2010; BoHaSA 2011; Daling et al. 2014), and these processes occur simultaneously and have feedbacks that induce both a chemical and physical change to the properties of the oil. The relative importance of each process is time dependent, with the physical and chemical processes transforming the properties of the oil from the moment the oil makes first contact with seawater. Consequently, an understanding of the way in which these multi-faceted weathering processes interact temporally and spatially is essential when modelling the changing characteristics of an oil during the lifetime of a slick at sea (e.g. ITOPF 2014).

The fate and behaviour of oil spills are exceedingly complex and it is fundamentally important to understand the processes involved with respect to the Arctic conditions encountered, and how these processes interact both temporally and spatially to alter the properties and behaviour of oil with time. By doing so, algorithms can be developed so that models can predict how an oil spill will weather and drift over time, given a specific set of environmental conditions, and a knowledge of the chemical composition of the oil.

**Fig. 2:**
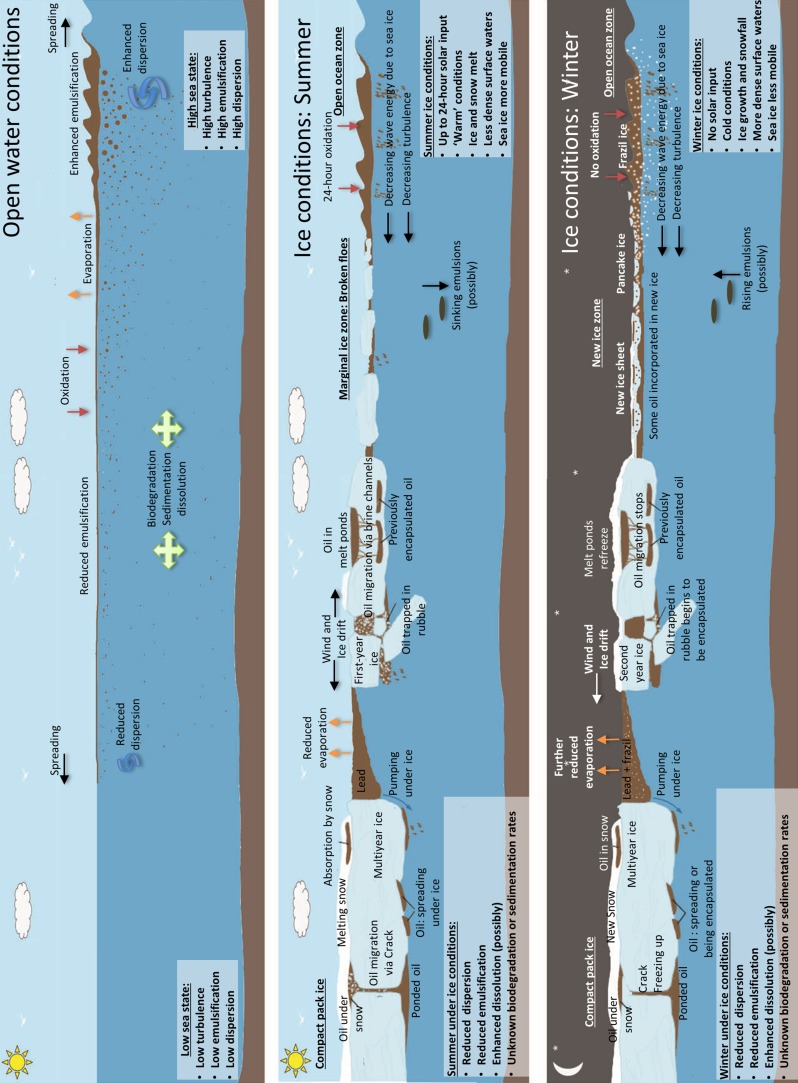
Sequence of the main oil–ice interaction and weathering processes in (top) open water conditions, (middle) ice conditions in summer and (bottom) ice conditions in winter (based around original figure by Bobra and Fingas (1986) and Wilkinson et al. (2013)). The main environmental factors influencing (top) ‘warm’ open-water weathering processes (besides oil composition) include amount of wind and wave energy; (middle) Summer sea ice weathering processes include amount of open water, wind-wave conditions, oil migration processes, wind herding in leads and the movement of ice; (bottom) Winter sea ice weathering processes include ice type, amount of daylight, drift of ice, encapsulation of oil, in new ice types as well as at the bottom of older ice * Not shown in this figure is a further scenario, specific to fast ice, whereby heat and/or gas contained within the oil, from rising blowout plume, inhibits ice formation and keeps an area of open-water ice free from late summer, and throughout the following winter (Lewis 1976)

There are significant and important differences between the transport and weathering behaviour of oil in the presence of ice versus open water. Cold temperatures and limitations on spreading due to the presence of sea ice decrease evaporation rates significantly. The absence of breaking waves reduces both emulsification and natural entrainment of oil droplets into the water column, and the spread of oil under ice is very different to its open-water counterpart. A schematic of the main oil–ice interaction and weathering processes for open water conditions, summer ice conditions and winter ice conditions is shown in Fig. 2.

Experimental observations performed in both the field and laboratory clearly show the importance of understanding the properties of oil that has been spilt in cold environments (Sørstrøm et al. 2010). However, many of these weathering experiments concentrate on oil that is in contact with the atmosphere, and further research is needed to better understand the weathering of oil that is trapped below the sea ice. This is extremely difficult to perform in the Arctic, as it requires the long-term monitoring of specific spills of different oil types in the marine environment. Long-term, controlled tank experiments are therefore extremely valuable as they can give insights into some of the natural weathering processes, but they cannot, and should not be expected to replicate the complexity of the marine environment.

Other areas that need further investigation include biodegradation processes in Arctic waters, quantifying the relative importance of the different natural weathering processes at different times of the sea ice growth and decay process, and the parameterisation of the vertical migration of oil through sea ice during the summer months. As with all of these types of experiments, it is important that these and future results continue to be made freely available to the community so that new parameterisations can be developed and tested to ensure we are able to accurately predict the weathering as a function of time and space. When doing so, it is particularly important to consider the impact of Arctic change on these processes.

### Modelling an oil spill in ice-covered waters

The origin of oil spreading models stretches back to the 1960s with the pioneering work of Fay (1969). Soon after these seminal papers, research and modelling began to look at the problem of the spreading of oil under solid ice. Work began in the early 1970s with models by Glaeser and Vance (1971) and Hoult et al. (1975) as well as a large body of experimental work linked to the Beaufort Sea Project in the 1970s (Milne and Herlinveaux 1977). Wadhams (1976b) used ice topography data from the Beaufort Sea to estimate oil containment by ridges. Over the next few decades, the sophistication of these models continued to improve, including the coupling of ice-hydrodynamic-oil models that have enabled sea ice dynamics and oil spill dynamics to be integrated with oil dispersion and weathering algorithms (e.g. Skognes and Johansen 2004). Further advances include the introduction of new sea ice rheologies and validation techniques to improve sea ice modelling in view of oil spill trajectory forecasting (Olason et al. 2016). More recently, a numerical study has been performed investigating the effects of a warmer climate on the fate of oil released in a spill in the Arctic (Nordam et al. 2017). New applications of chaos theory, known as Lagrangian Coherent Structures, are being applied to drift problems such as oil spills. These new analyses of currents and winds show that environmental fields can be analysed to determine areas spills are blocked from reaching (Allshouse et al. 2017) or to predict locations of future rapid changes in an oil spill earlier than traditional oil spill models show any changes (Olascoaga and Haller 2012).

With respect to oil under sea ice itself, Fingas and Hollebone (2003) suggested that all existing oil-under-ice models are inadequate because they are unable to represent the complexity or uniqueness of the bottom topography of sea ice. Wilkinson et al. (2007) addressed this concern by obtaining accurate in situ data on the three-dimensional shape of the underside of first year sea ice through the use of an autonomous underwater vehicle fitted with an upward-looking multi-beam sonar. By coupling these data to a simple oil spill model, a realistic appraisal of the potential oil holding capacity of first year sea ice was achieved.

One of the main advantages of numerical simulations is the flexibility they allow in understanding a complex system. However, it is easy to fall into the trap of trusting the output of a model without question. The age-old cliché that “a model is only as good as the parametrisations and the input data” remain true. Therefore, a clear understanding of the limitations of any model is essential. Whilst many of first-order processes that govern the weathering and trajectory of oil within an Arctic marine environment are known and parametrised, the uncertainty associated with the output from these models is presently unquantified.

The accuracy and uncertainty of an open-ocean oil spill model can be validated by analysing data from various real oil spills from shipping accidents or blowouts that have occurred under different weather and oceanic conditions. For example, modelled oil trajectories can be validated against daily spread of oil as detected by satellite or airborne sensors. Few such datasets are presently available for the ice-covered seas, the ‘best’ in recent years being the spill from the loss of the *MV Runner 4* in the Gulf of Bothnia (Wang et al. 2008) and the *MV Godafoss* in Norway in 2011 (Broström et al. 2011). There is a real dearth of in situ data for the validation of oil trajectory and fate models for the ice-covered seas. As a result, there is a real need for a limited, but controlled in situ oil spill campaign to gather an openly available dataset for oil spill modelling teams to validate their model simulations. This publicly available benchmark dataset could be used to identify discrepancies between models, enable parameters in a model to be tuned, and allow for new algorithms and parametrisations to be developed as and when needed. A first step along this road is for the modelling and observational communities to identify what set of parameters are most important to measure during an in situ campaign, and at what temporal and spatial resolution.

## Oil detection and monitoring

The detection and monitoring of an oil spill in ice-covered seas has made significant advances over the past decade. For example, recent tests under laboratory-grown sea ice have revealed a number of sensors that have the ability to detect oil that is located under or encapsulated within the sea ice (Pegau et al. 2016). Even so, the presence of ice and the other environmental conditions that prevail in the Arctic can limit the feasibility, or effectiveness of, the various oil spill detection and monitoring techniques that are available. All oil detection techniques and technologies have their own specific advantages and limitations as to how they can be applied in the Arctic environment, particularly ice-covered seas. The remote detection of oil spills in ice-covered seas can however be divided into three sections:

1. Detecting oil with sensors mounted above the ice cover;
2. Detecting oil with sensors mounted on the ice cover; and
3. Detecting oil with sensors mounted below the ice cover.

We review these and comment on the reliability in detecting oil, the spatial coverage and the time taken to deploy and conduct a survey of a spill site. A number of review papers are available (Fingas and Brown 1997; Brekke and Solberg 2005) that describe the available remote sensing technologies for detecting oil spills in open water. Recent reviews for ice-covered seas include Wilkinson et al. (2013) for detecting oils spill from underneath the ice, and Puestow et al. (2013) for detecting oil spills from above the ice.

### Sensing from above the ice cover

Attempting to detect oil on, within or under ice presents many technological challenges which are not easily overcome. The past 20 years have seen major advances in satellite and airborne sensor technologies that allow study of the Earth’s surface at a number of radiative frequencies suitable for classifying the type of surface and allowing regular mapping of a number of parameters including, but not limited to, land use, sea ice cover and ocean colour.

Whereas in the past there was limited availability of Earth observation data, a number of organisations have promoted free public access to satellite data including National Aeronautics and Space Administration (NASA) in the USA and the European Space Agency (ESA). This has made it easier to research into new methods of detecting particular surface types, making algorithms available for the operational community to start producing services covering areas of interest to the community in general. In Europe, the Global Monitoring for Environment and Security (GMES) programme, sponsored first by ESA and now taken up by the European Commission (EC) under the name “Copernicus”, has led to the development of new services with the Sentinel series of satellites.

#### Satellite remote sensing

Remote sensing from satellites can play a role, both in the continuous monitoring of spills, and in aiding the response efforts. Whilst remote sensing of sea ice and the detection of oil spills using satellites are both widely studied, there has been little work to investigate the two in combination. A particularly tricky problem for the Arctic is how to detect an oil spill if it is from an unknown incident or illegal activity such as discharge.

Both optical and active microwave sensors have been the focus of some preliminary studies on oil spill in sea ice detection. While optical sensors have shown some success in detecting oil spills on top of the sea ice, their use in routine monitoring is limited by the clouds and darkness (polar night) prevalent in Polar Regions. Active microwave in the form of imaging Synthetic Aperture Radar (SAR) is the preferred approach for the Polar Regions as it can see through clouds, and is not influenced by the presence or absence of sunlight. Although SAR is proven in detecting oil spills in open water, its use for detecting oil spills in ice-covered waters requires more research. An in depth investigation of polarimetric SAR techniques using the new generation of satellite sensors that are now becoming routinely available could be particularly valuable.

SAR is capable of mapping objects down to a few metres size, and can be targeted on the site to provide monitoring of individual ice floes and possible spill mapping. In May 2009, a combination of industry and research partners undertook fieldwork off eastern Svalbard as part of a Joint Industry Program (JIP) project on oil spill contingency for Arctic and ice-covered waters (Babiker et al. 2010). This acquired different types of SAR imagery, including Envisat ASAR, Radarsat-1 and -2 and COSMO SkyMed, to assess oil in ice detection for single- and dual-polarisations. There were no fully polarimetric image acquisitions. Ice conditions were 7–9/10ths concentration with 5–30 m floe sizes and 15–35 cm of snow cover. The study confirmed that the detection of oil in ice-infested waters is hindered by the formation of new ice (grease ice) that also dampens waves, and by low speed winds, as both these phenomena have the same SAR signature as oil on the seawater. They also concluded that detection is improbable when ice concentrations are moderate to high (greater than 4/10ths), and that small spills due to spreading within pack ice cannot be detected. Further studies are in progress to resolve this issue using full polarimetric SAR (e.g. Brekke et al. 2014), and with the use of near-surface scatterometer instruments in order to gain experience with possible space-borne SAR response (Firoozy et al. 2017; Petrich et al. 2017).

#### Aircraft

Aircraft can carry the same types of sensor as satellites, and their closer proximity to the sea ice surface allows much higher imaging resolutions. Whilst it is easier to target an area using aircraft, they can only cover a smaller area and take a longer time to do so, than satellites. Aircraft remote sensing is a specialised field, with a limited number of aircraft operators, especially in the polar regions. Therefore, the available aircraft tends to have a fixed complement of sensors, and adding new ones for specific task requires additional time, and in some instance special certification, that may not be available if a quick response is required. Baschek (2007) provides a review of the different types of available sensors, and how these could be integrated onto a surveillance aircraft. Airborne sensors such as SLAR (Side-Looking Airborne Radar) are successfully utilised for locating oil, and UV (ultra violet), IR (infra-red) and hyperspectral imaging sensors are used in determining the total extent of an oil slick and the relatively thick and thin oil layers within an oil slick (Puestow et al. 2013). It is important to reiterate that all these techniques are dependent on the ice concentration, and Arctic weather conditions, such as strong winds, reduced visibility (i.e. fog, drifting snow, low cloud base) and icing, can preclude flight operations.

Generally speaking, the larger the aircraft, the greater the range, endurance and different types of sensors that can be carried. Manned aircraft provides the greatest flexibility, but Remotely Piloted Aircraft Systems (RPAS) are beginning to become more widely available and their ease of deployment, once regulatory hurdles are overcome, may in future make it possible for them to take on more oil spill response work (Mulac et al. 2011). Because of the limited sensor payload and endurance of RPAS, but reduced space required for storage, they are suitable for being always on standby at an operations site.

#### Surface vessels

Aside from visual observations by trained observers, the radar systems installed on ships, typically X-band (8–12 GHz), are good at detecting ice features such as ice edges and ridges and have been proven in open water oil spill detection. In Norway, the Norwegian Clean Seas Association for Operating Companies (NOFO) has 14 ship-based radar systems, produced by MIROS^1^ in operation. The radars collect up to 128 scans of a target area and then use processing algorithms for oil detection. The range of detection is about 3 km from an antenna height of 18 metres (Dickins and Andersen 2009). Other systems available include the SeaDarQ system^2^ from the Netherlands, and the Canadian Rutter Sigma S6 ice detection radar.^3^ Whilst shipboard radar systems can detect oil on open water, their use on oil spills within ice-covered waters remains unproven.

#### Buoys

Buoys are not normally used to carry sensors that can detect oil, although systems are being developed through the EU funded GRACE programme.^4^ However they are very commonly used to monitor ice drift in near real time, and so can be deployed at a spill location so that the ice can be tracked for subsequent processing by oil spill clean-up teams.

### Sensing on ice

The remote sensing of oil using on or near ice technologies is limited to a handful of systems, the most positive being penetrating radar (GPR), and dogs. On-ice systems offer very limited area coverage and are time consuming. Safety aspects of having personnel on ice are also a consideration.

#### Ground Penetrating Radar (GPR)

GPR systems operate in the 500 MHz to 1 GHz frequency range. Whether that can penetrate all the way through the sea ice is dependent on ice thickness, the temperature of the ice, and the distribution of brine within the ice. Studies have shown that GPR can detect oil layers of about 1–3 cm thickness, on ice but buried beneath snow, and trapped in or under relatively smooth ice (Bradford et al. 2008, 2010). The capacity to detect oil trapped within or underneath ice depends on the properties of the ice and overlying snow. Snow normally has a very low electrical conductivity, thus allowing radar propagation, whilst sea ice has a much higher conductivity (> 10–2 S/m) that varies substantially both laterally and vertically (Morey et al. 1984), and can exhibit a high degree of anisotropy due to preferred crystal alignment (Kovacs and Morey 1978; Nyland 2004). These characteristics affect the ability of GPR to penetrate into the ice and detect oil. It is more challenging to obtain good GPR surveys from warm, young year ice, with its higher proportion of brine pockets. Processing of data from GPR is computationally intensive, and some results can be ambiguous even for a trained operator. Although it is possible to map oil within and under ice using this method, the on-ice method is time consuming, and thus efforts are being made to establish a helicopter-mounted GPR system. Tests by Bradford et al. (2010) using a helicopter based, 1000 MHz GPR system, showed that it was able to detect a 2-cm-thick oil film located between snow and sea ice based. More recent tests confirmed that the GPR was able to detect encapsulated oil in an airborne mode when the ice was cold (Pegau et al. 2016).

#### Dogs

A less technological approach is detecting oil by specially trained dogs. Sniffer dogs are already used to search out explosives and drugs, and their use for detecting oil buried under snow on sea ice has been field tested as part of the Oil in Ice—JIP (Brandvik and Buvik 2009; Dickens et al. 2010). This study found that the dogs were able to pinpoint the locations of very small oil spills that had been left for a week, determine the dimensions of larger oil spills consisting of clusters of small spills and indicate the direction to larger spills up to 5 kilometres away upwind.

### Below ice

The detection of oil spills from under the ice is probably the least studied technological sector. This has been due to most oil spill detection studies being concerned with open water, where an underwater approach has not been necessary. The use of underwater vehicles (manned submarines, or unmanned such as Remotely Operated Vehicles (ROVs), Autonomous Underwater Vehicles (AUVs) and ocean gliders), in the Polar Regions has a long history. However, it is only in the past decade or so that technology has advanced to a state where ROVs, AUVs, and ocean gliders are a practical proposition for under-ice remote sensing (Wilkinson et al. 2006, Lee et al. 2017). However, most operators of these vehicles are not experienced with under-ice operations. Unless a nuclear military submarine fitted with the correct sensors is available, which is unlikely, then oil spill response is limited to ROVs, AUVs and possibly gliders. These usually require a ship or personnel on ice to support operations. Generally, smaller ROVs and AUVs can be operated by personnel on ice, with larger vehicles requiring support infrastructure such as a ship with heavy lifting equipment. Most underwater vehicles, due to their reliance on battery power or an umbilical tether, suffer from limited range and mission endurance. Depending on the sensor payload, sampling strategy and mission priorities gliders, being a slow moving buoyancy driven device, can have an extended presence in a region. Recharging the batteries is often slow and requires taking the vehicle out of the water. A recent review of sensors that have the potential to detect oil located under sea ice can be found in Wilkinson et al. (2013). These include acoustics (sonar), laser fluorescence, camera systems, radiometers and multispectral sensors, and mass spectrometry. Testing of these sensors by Pegau et al. (2016) suggested that all the above-mentioned sensors showed, under certain conditions, an ability to detect oil below or encapsulated within the ice. For example, the cameras and radiometers could detect oil at various depths in the ice, whilst the laser fluorescence and acoustic sensor was able to detect oil below the ice as well as encapsulated oil (within 6 cm of the ice bottom). Importantly, the acoustic systems were able to accurately measure the thickness of oil below the ice, a particularly valuable trait for oil recovery operations.

### Summary of oil detection and monitoring

In summary, the detection of oil spills by sensors that can cover large areas quickly and accurately are preferable. For open water spills, this is achievable with satellite or airborne sensors, and results suggest that these techniques are expected to work for oil spill detection in very open drift ice, up to 3/10ths concentration. In heavier ice concentrations, the sensor performance and detection capabilities of satellite and airborne sensors are less robust and in some cases unknown.

As the concentration of ice increases, the likelihood of oil being located under (or within) a sea ice cover also increases. The detection of oil under sea ice is a difficult task, but investment and research in this field have delivered a number of sensor technologies that have the potential to detect and map oil under or within sea ice (Puestow et al. 2013; Wilkinson et al. 2013; Pegau et al. 2016). However, our literature review suggests that very few, if any, are truly operational at present. The advantages and limitations of the most promising technologies to detect oil under different sea ice, oceanographic and meteorological conditions need to be fully established. Once suitable technologies have been identified, it is essential that investment continues to ensure that operators are familiar with the routine deployment of these instruments under different environmental conditions, and proficient with the accurate and timely interpretation of resultant data.

## Oil spill response techniques

Methods that have been found to be effective in responding to oil spills at sea in temperate climates are (i) mechanical recovery with booms and skimmers, (ii) dispersant use and (iii) in situ burning. Each of these methods has particular capabilities and limitations that make it more or less suitable for responding to specific oil spill situations. The methods that would be feasible or effective for spills of oil in ice-covered waters vary depending on the seasonal ice and other conditions. The behaviour of oil spilled in cold, ice-covered waters is governed largely by the ice concentration in the case of broken ice and the process of encapsulation and subsequent vertical migration in the case of solid ice. For example, if oil is spilled under ice in the spring (after May), the oil might not become encapsulated in the ice due to insufficient new ice growth before seasonal melting commenced (Buist et al. 2008a). Conversely, a spill occurring just prior to or during freeze-up (Lewis et al. 2008) may become rapidly incorporated in ice, such that response efforts could include a combination of oil recovery and ice tracking and monitoring operations.

Each season presents different advantages and drawbacks for spill response:

- During the summer open-water season, except for remoteness, oil spill response will be as in temperate waters;
- During freeze-up and ice growth, drifting ice and limited site access will restrict the possible response options;
- Mid-winter, with long periods of darkness and intense cold, provides in the case of fast ice, a stable ice cover that not only naturally contains the oil within a relatively small area, but also provides a safe working platform for oil recovery and transport. The opposite is the case for drifting ice, which has the ability to transports irregular slicks of entrapped oil to regions well beyond the spill site, and thus can contaminate a vast area of the Arctic Ocean (Wadhams 1989, 2012);
- During the thaw, breakup and final melting of the ice, the response to oil spills in moving pack ice is likely to be more limited due to the changing nature of the ice pack and the need for ice-strengthened vessels.

Nuka Research and Planning Group, LLC (2007) points out that the range of ice conditions that may be encountered in the Beaufort Sea is an important factor when determining what types of technologies are “appropriate and reliable” for oil spill response and recovery.

### Mechanical containment and recovery of oil in ice-covered waters

The purpose of conducting mechanical containment of spilled oil at sea is to limit the spread of spilled oil by containing it within a boom and then recovering the oil from the sea surface and onto vessels for subsequent disposal. The removal of oil from the marine environment will reduce the damage that could be caused to ecological and socio-economic resources. Mechanical recovery has been demonstrated to be a potential strategy in solid, fast ice (Allen and Nelson 1981). The advantage of mechanical containment and recovery is that the spilled oil is removed from the sea surface and is prevented from subsequently drifting to the shore. One of the main disadvantages of mechanical containment and recovery at sea is that it can be a slow process; it has a low ‘encounter rate’ and the oil can spread faster than it can be recovered. A summary of the feasibility of existing and potential future equipment to improve effectiveness of mechanical recovery in the Arctic can be found in North Slope Spill Response (2015), and the NRC study (2014). A major problem is that the material recovered may have a low density of oil, comprising mainly oiled snow and ice, yet has to be stored safely in its entirety and disposed of. Suitable storage/melt facilities on the scale needed do not exist in the Arctic. One major oil company, for instance, envisaged permanently stationing a large tanker in the Arctic to receive oiled snow and ice from a possible spill.

When responding to an oil spill in Arctic conditions, the first step is to identify the oil’s physical properties, particularly the pour point. If the pour point is 5–10 °C above the water temperature, there is a strong possibility that the oil will be solid. Nets and other collection devices may be required for recovery. If the pour point of the oil is below the water temperature and if currents and wind conditions allow, then booms and skimmers may be applicable for use.

#### Booms

It is obviously not feasible to use a floating boom to contain spilled oil if there is total ice coverage or encapsulation within the ice itself. The oil will either be on top of the ice, possibly covered by snow, or on the underside of the ice. Partial ice cover will act as a series of naturally occurring booms, limiting the spread of the ice in certain areas. If sea ice coverage is greater than about 6/10th, the ice itself can potentially serve as a natural containment barrier (Dickins and Buist 1999).

The basic problem about using booms to contain spilled oil in partially ice-covered waters is that the boom contains floating ice as well as floating oil. The feasibility of using booms is therefore related to ice coverage. Ice concentrations as low as 1/10th negatively affect large, open towed-boom systems. Attempts to tow a boom from a vessel to contain spilled oil will result in a lot of ice being ‘captured’ within the boom. This will put a strain on the boom, tear the flotation chambers and possibly break the cables within the booms. There are a number of types of booms available for use in low coverage concentrations of ice in ice-covered waters (DeCola et al. 2006). Ice booms also have the capability to assist other mechanical recovery systems by providing an ice-free environment, and in separating oil from ice (Abdelnour and Comfort 2001; Abdelnour et al. 2001). The collection of spilled oil in booms is feasible, with suitable techniques and reduced effectiveness.

Recent advances in technology have been made to extend the capability of ice booms, adapting technology that had been in use for several decades to protect water intakes upstream of hydroelectric power plants into a countermeasure for oil spill response. Techniques to deflect and separate oil from ice on the sea surface, such as using prop wash or pneumatic bubblers, may enable mechanical systems to encounter and recover oil at higher rates in the presence of drifting ice.

#### Skimmers

The most appropriate skimmers for ice-covered waters are the oleophilic rope mop and brush skimmers. These skimmer types are preferred because other skimmers will quickly become clogged with smaller pieces of ice. Even very low concentrations of ice seriously affect the performance of most skimmer systems through plugging and bridging. Skimmers work best when positioned in open water and in leads between ice pieces.

Two programmes that have developed mechanical oil recovery systems for deployment in ice-infested waters are (i) the Mechanical Oil Recovery in Ice-Infested Waters (MORICE) project (Jensen and Mullin 2003) and (ii) the Lamor Oil Ice Separator (LOIS) (Minerals Management Service 2008).

Solsberg (2008) noted that there have been several recent advances in mechanical recovery systems for spill response in Beaufort Sea spring breakup or fall-freeze-up seasons. However, there can still be severe limitations during deployment due to ice-processing challenges, extreme weather (freezing) conditions, and changing conditions in the ice itself.

### The use of oil spill dispersants in ice-covered waters

The purpose of using dispersants on spilled oil is to transfer the oil from the sea surface into the water column. This is done to prevent the spilled oil from drifting and eventually contaminating the shoreline. When dispersants are sprayed onto the spilled oil on the water surface, the surfactants in the dispersant greatly reduce the interfacial tension between the oil and the seawater. This enables the prevailing turbulence of wave/wind action to convert a larger proportion of the spilled oil volume into droplets that are small enough to be rapidly diluted into the water column (NRC 1989, 2005). Dispersing the oil as very small droplets in the water column enables naturally occurring hydrocarbon-degrading microorganisms to substantially biodegrade the oil, leaving a small proportion of recalcitrant residue (Prince et al. 2013).

Concerns expressed about dispersant use often revolve around the potential effects that could be caused by increased exposure of marine organisms to dispersed oil and the partially water-soluble chemical compounds from the oil. The concentration of dispersed oil, and the compounds from the oil, in the water rapidly decreases as the oil is diluted into the water column. The oil in the water column will be rapidly diluted to concentrations below the toxicity threshold limits. The exposure to concentrations that are possibly high enough to cause negative consequences to marine organisms is brief and in a limited volume of water. However, if large quantities are involved, such as injecting dispersants directly into the oil–gas plume rising from a blowout site in order to prevent a slick from forming at the surface, the toxicity dangers must be assessed carefully (NRC 2014).

Overall, the potential negative effects of dispersant use, such as the possible localised impact on marine organisms needs to be balanced against the possible positive effects such as avoiding serious damage to coastal and sea surface resources. Dispersant use in the Arctic, like any oil spill response method, should be subject to a Net Environmental Benefit Analysis (NEBA) (IPIECA-IOGP 2015) (sometimes referred to as Spill Impact Mitigation Assessment, SIMA). This process assesses the relative impact mitigation potential of candidate response options, in order to choose those that will most effectively minimise the overall consequences of a spill.

Dispersant use could be a response to oil on the sea surface amongst broken ice. The dispersant would need to be sprayed onto the oil to achieve the recommended treatment rate of around a DOR (Dispersant to Oil Ratio) of 1:25. The mixing energy to cause initial dispersion of the dispersant-treated oil is normally provided by breaking wave action in the open sea, but this will be limited in the presence of ice because ice dampens the waves. The mixing in the upper layer of the water column that dilutes the dispersed oil will be less rapid when ice is present. Additional mixing energy supplied using ship’s thrusters might be required (Spring et al. 2006). Brandvik et al. (2006) report that dispersants can be a suitable oil spill response in Arctic waters in open water and up to 5/10th ice cover. In a review of dispersant effectiveness under Arctic conditions, Lewis and Daling (2007) identify factors, such as the presence of sea ice and colder temperatures, that may reduce the effectiveness of dispersant applications. Dispersants became less effective when the oil is above a viscosity of approximately 10 000 cP (centipoise) or more (Lessard and DeMarco 2000). A lower temperature causes a higher oil viscosity. However, low temperature and the presence of ice also restrict oil spreading and inhibit oil weathering, such as evaporative loss of the more volatile oil components to the air and the formation of water-in-oil emulsions (Fingas 2008). The time ‘window of opportunity’ for effective dispersant use can be significantly longer with partial ice coverage than in open water in a temperate sea. Results from tests conducted at the National Oil Spill Response Research and Renewable Energy Test Facility (formerly OHMSETT) using four Alaskan North Slope crude oils and two dispersants found that the dispersants were more than 90% effective at dispersing fresh and weathered forms of the oils under cold weather conditions (Mullin et al. 2008; Belore et al. 2009).

As low prevailing temperatures do not preclude dispersant use, the potential effects need to be considered. Concerns over the sensitivity of Arctic marine species to dispersed oil have sometimes been expressed, but studies of a wide range of Arctic species indicate that they are no more sensitive to dispersed oil than their temperate cousins (Bejarano et al. 2017). The balance between the consequences of short-term, localised exposure of marine organisms to dispersed oil and the potential longer term benefits of dispersant use will need to be made using NEBA/SIMA.

A particular aspect of Arctic dispersant use may warrant some further study. After dispersant use, a small proportion of the oil volume may resurface because the oil droplets are not small enough to be maintained in the water column. This resurfacing oil could surface under sea ice. Where the oil droplets would then be in close contact with ice algae. Similarly, careful consideration using NEBA would be required for subsea dispersant injection as a response to a subsea blowout, as occurred at the Deepwater Horizon oil spill. Large quantities of dispersed oil would be produced in the water column and some fraction would rise to the sea surface and could become trapped under ice.

Considering the longer term fate of the dispersed oil, a common misconception is that the low temperatures of Arctic seawater will slow biodegradation of oil, either by directly affecting the microbes, or by altering oil properties such as viscosity and pour point. In fact, measured biodegradation rates are remarkably similar with half-lives of 17 days at 5 °C (Brakstad et al. 2015), 18 days at −1 °C and 14 days at −1.7 °C (Garneau et al. 2016). Once oil has been dispersed into the water column, it will be biodegraded reasonably promptly, with a ‘half-life’ of a few weeks. One note is that the “propane jumpstart” to biodegradation during the Deepwater Horizon oil spill (Valentine et al. 2010) is not seen in water from a pristine Norwegian fjord (Brakstad et al. 2017), which indicates that areas without natural oil seeps may show much slower biodegradation rates than seen in the Deepwater Horizon oil spill. A recalcitrant, non-biodegradable residue will remain, but the recalcitrance to biodegradation and dispersion over a wide area suggests that this will have little environmental impact and minimal toxicity.

The main environmental characteristics of the Arctic, the low winter temperatures, the long periods of darkness during the winter, the remoteness, and the presence of ice and snow for much of the year, pose challenges to the operational use of dispersants, as they do to other methods of oil spill response. Results from studies conducted over the last 35 years indicate that dispersant use in the Arctic is a feasible response to spilled oil.

### In situ burning in ice-covered waters

Some of the earliest in situ burning activities were laboratory, tank and field studies conducted in the 1970s associated with drilling in the Canadian Beaufort Sea (Potter and Buist 2008). A series of successful Arctic field experiments in the 1970s and early ‘80s were largely responsible for helping in situ burning become accepted as an effective oil recovery strategy in situations involving spills in ice-covered waters.

Research and development efforts intensified in the years following the “Exxon Valdez” spill in 1989 to improve fire-resistant boom design, refine operational procedures and to resolve issues associated with air pollution from burning. These research efforts culminated in an international, multi-agency research burn in August 1993, known as the Newfoundland Offshore Burn Experiment or NOBE (Fingas et al. 1995). The experiment verified that in situ burn operations can be conducted safely and effectively with burn efficiencies exceeding 90%. Brandvik et al. (2010) report in situ burning efficiencies ranging from 50 to 90% in field tests (during about 7/10th–9/10th ice coverage), and in meso-scale laboratory experiments in a wave tank under varying ice coverage conditions (no ice, 5/10th and 9/10th ice coverage).

One of the key challenges to the effectiveness of in situ burning is maintaining sufficient thickness of oil to sustain a burn. The minimum ignitable thickness of a fresh crude oil slick on water is about 1 mm, whereas for aged, unemulsified crude oil the minimum thickness is on the order of 2–5 mm (Potter and Buist 2008). Emulsification is an important process influencing the effectiveness or the response window of opportunity for use of in situ burning, because the oil in the emulsion is not able to reach a temperature in which it is able to burn until the water is first boiled off (Potter and Buist 2008).

Oil may be more difficult to ignite at low temperatures but once burning begins, it will continue regardless of ambient temperature. The effectiveness of in situ burning can be affected by weather and sea-state conditions, but ice coverage is also a very important factor. At ice coverage exceeding 7/10th in situ burning can be conducted without any mechanical containment systems, as the ice provides a natural barrier to restrict the movement of oil across the water surface. At ice concentrations less than 3/10th, open-water in situ burning may be feasible (Brandvik et al. 2006; Potter and Buist 2008), including the use of oil containment with a fire-resistant boom. Ice concentrations of 3/10th–7/10th are considered to be the “most difficult from an in situ burning perspective” (Juurmaa 2006). These ice concentrations are high enough to impede the effectiveness of mechanical containment systems, but too low to serve as a natural containment barrier for the oil (Brandvik et al. 2006; Potter and Buist 2008).

In addition to the ice coverage, the type of ice present can alter the effectiveness of in situ burning (S.L. Ross Environmental Research, Ltd. et al. 1998). Conducting in situ burning in pack ice during breakup may be more effective at removing spilled oil than when there is a similar amount of ice coverage during the fall freeze-up, because the fall freeze-up generates significant amounts of slush ice that can impede containment of slicks (Potter and Buist 2008).

As reported by Buist et al. (2013), the behaviour of oil and sea ice largely dictates whether in situ burning is possible for a given spill. Generally, in situ burning may be the preferred response strategy for oil spills in broken ice where it is not safe to work in or on the ice. In situ burning can also be the preferred technique for dealing with spills on ice and snow-covered surfaces; oiled snow with as much as 70% snow by weight can be burned. In situ burning is also a possibility for oil released through brine channels into melt pools in the ice during spring thaw. Burning oil at sea generates copious amounts of smoke because the basic layout of a pool-fire restricts the access of air to the base of the flame. Moderate wind speeds help combustion. Not all the oil will burn and a viscous, high-density tarry residue, perhaps 5% of the original volume of the oil, will remain. The residue from an in situ burning may float on water or sink, depending on the oil type and the extent of the burn.

### Chemical herders used in conjunction with in situ burning

Chemical herders, sometimes referred to as oil collecting agents, are chemicals applied to the water surrounding an oil spill in order to thicken the spill, without the need for mechanical containment, to a point that it can sustain a burn (Buist et al. 2008b; Minerals Management Service 2008). Chemical herders constitute an oil spill countermeasure that can be used in conjunction with in situ burning (Sørstrøm et al. 2010).

Chemical herders have been available for several decades (Buist et al. 2008b), but not used extensively offshore to date because they are only effective under largely calm conditions (S.L. Ross Environmental Research Ltd. 2010); however, their use within the ice-covered seas is presently not well constrained. Reviews on the state-of-the-art of oil spill countermeasures, such as that by D.F. Dickins Associates Ltd (2004), identified chemical herder behaviour in ice environments as a knowledge gap and subsequent research activities (Minerals Management Service 2008; Buist et al. 2008a; Interagency Coordinating Committee on Oil Pollution Research 2009) focused on the potential utility of herders in responding to oil spills in cold waters, and particularly in ice-covered waters (SL Ross and Danish Centre for Energy and the Environment 2015).

Two full-scale burn experiments involving the use of chemical herders were conducted in the offshore of Svalbard, Norway (Minerals Management Service 2008; Pew Environment Group 2010). One large-scale experiment with chemical herders was carried out on a free-floating crude oil slick in low (1/10) ice coverage as part of the JIP Oil-in-Ice effort in 2008 (Sørstrøm et al. 2010).

One of the formulations used in recent studies of chemical herders in cold-water conditions is the U.S. Navy cold-water herder formulation (Buist et al. 2008a, b; Buist 2010). This herding agent was successful in producing slicks in excess of 3 mm and in significantly contracting oil slicks in the presence of ice (Buist 2010). New formulations of chemical herders are under development and testing (Buist et al. 2010).

### Summary of oil spill response techniques

In summary, there have been many research programmes into methods of oil spill response in ice-covered waters including containment and mechanical recovery, burning, bioremediation and enhanced dispersion. Some oil spill response methods that would be feasible or effective in open water condition are of limited value in ice-covered waters. Furthermore the effectiveness of in-ice response methods varies depending on the ice, ocean and meteorological conditions. Essentially each season presents different advantages and drawbacks for spill response. A review of the literature suggests long-term investment in this field has been made and there are a number of possible techniques available. However, it was difficult to establish exactly what range of environmental conditions each system could operate in. There needs to be a focus on establishing the efficiency and effectiveness of each system under a range of ice conditions and weather conditions, especially considering drastic climate changes occurring in the Arctic today and predicted for the future. Furthermore, any of the human-intervention techniques such as burning and dispersants remove oil from the ocean surface, but their impact on the Arctic ecosystem, and in the case of burning the impact to the atmosphere, is presently unknown. Studies need to be performed to quantify their impact of the marine environment and how this impact varies both temporally and spatially.

## Suggestions and future needs

We have shown that understanding the impact, response, and potential consequences of an oil spill in the Arctic marine environment is both a research and operational challenge that requires expertise from a wide spectrum of individual specialties. It is also clear that the extreme range of environmental conditions that can be present in the Arctic marine environment poses a challenge to any oil spill response. The past 40 years of Arctic oil spill research have provided a solid baseline knowledge regarding the fate of oil, as well as an understanding of the operational solutions and techniques needed to detect and recover the oil. Whilst our knowledge-base is broad one can legitimately ask:

- Is our knowledge of the fate of different oil types in cold seawater and/or sea ice-covered environments sufficient to develop effective spill response and remediation strategies for today? And are these strategies sufficiently robust to accommodate the predicted climate-driven changes in the region over the coming years?
- Do we have the operational and logistical capability, technology and command structure to viably mount an oil spill contingency operation in any season?
- Are the present policies, regulations and best practice approaches appropriate for the Arctic marine environment?
- Do we have a baseline understanding of the Arctic marine environment in order to accurately predict the impact of an oil spill on the short (less than a year) to medium time (1–10 years) time frame?
- Do we have the capability to compare with reasonable accuracy the potential risks and potential benefits of Arctic oil exploration/production to support evidence-based decision making?

There are undoubtedly some gaps in our knowledge-base, which only can be filled through research, technological development, operational testing and refinement. We need to eliminate the cycle whereby intense periods of research are interspersed by long periods of much less activity. This approach can be detrimental as institutional memories begin to fade, built-up expertise can be lost, older experts retire and training of new experts is sporadic. This feast or famine approach should be replaced by long-term, strategic investment. Recently, this more sustainable approach to Arctic oils spills seems to have received some traction as we have seen focused investment in basic research by industry through the Joint Industry programme,^5^ as well as by governments such as the European Union’s ACCESS project,^6^ GRACE programme^7^ and others. Our ability to successfully execute controlled oil in sea ice experiments, as performed within ACCESS, is also improving through existing facilities such as the following:

- the Arctic Environmental Test Basin (AETB) at the Hamburg Ship Model Basin (HSVA),^8^
- the Cold Regions Research and Engineering Laboratory in the US,^9^
- the Churchill Marine Observatory in Canada, which is a major facility for the study of detection, fate, effects and mitigation of oil spills in ice-covered waters,^10^
- The SINTEF Ocean AS climate rooms with laboratory and meso-scale experimental facilities.^11^

Whilst structured laboratory experiments are vital in further developing our understandings of key processes, detection methods, and equipment testing, controlled oil release field trials are essential to achieve and maintain a credible state of readiness. At present, most government regulations prohibit the controlled release of oil, but occasionally permits are issued for research purposes.

Oil release experiments, particularly in the Arctic, need to be carefully planned and permitted so that the maximum amount of science can be performed, whilst minimising environmental effects. These well thought out controlled spills need to be designed for a wide range of ice conditions, oil types, and spill scenarios. Progress requires equal partnership with a diverse range of stakeholders, including local communities. In addition to these controlled ‘real world’ experiments other strategies need to be developed whereby we can semi-regularly perform quantifiable stress tests on different aspects of Arctic oil spill contingency planning, associated decision support systems, and the system as a whole.

At the current prevailing oil price, oil exploration and production in Arctic waters remain at low levels, as does Arctic shipping; however, the consequences of an accidental spill of oil (crude or processed) into the Arctic marine environment could be severe. Given these low levels of oil and shipping activity, we have a ‘window of opportunity’ to develop more robust solutions and protocols to meet the above-mentioned challenges. This needed research, and planning is not inexpensive, and will need continuous evaluation and refinement as science, data access and technology improve. No one can guarantee an accidental spill cannot happen, and no response method is risk free or completely effective. Therefore, to reduce the likelihood of an accident and accompanying consequences, we must have a comprehensive understanding of the issues involved, ensure best practices are followed, have a robust risk management framework, and have a responsive decision-making structure is in place.

## Conclusions

There are significant differences between oil spill response capabilities in open water and in ice-covered waters. Significant challenges also exist for spills occurring within different sea ice types, concentrations and seasons, all of which are strongly impacted by climate change. There is no getting away from the fact that the ice-covered regions are complex. With the renewed interest in the Arctic and with the pace of activity in the marine environment increasing, we cannot be complacent regarding oil spills in the Arctic marine environment.

Whilst it has long been recognised that the Arctic marine environment represents one of the most challenging areas in the world in which to work, a wealth of technical and operational expertise, experience, and know-how has been developed within industry, government, and academia. This knowledge also extends to oil spills in ice-covered seas. Nevertheless, there are deficiencies in our understanding that need to be addressed so that these gaps can be bridged and solutions found.

In order to comprehend fully our level of understanding and readiness to deal with an Arctic oil spill, field exercises that encompass a broad spectrum of sea ice, ocean and meteorological conditions will be necessary. Whilst a handful of controlled oil spills experiments have occurred in sea ice the past, new developments and techniques suggest that further controlled field trials are needed to evaluate and improve oil spill response capabilities and technologies. These exercises, whilst very challenging, should be encased within the realities of the climate-driven changes within the region.

While the long-term goal is to reduce our reliance on hydrocarbons, and thus reduce our global carbon footprint in line with the legally binding Paris Climate Agreement we must be ready to deal with an accidental spill in the Arctic now. There is urgency to this readiness evaluation as exploration, shipping, and in some instances production, in ice-infested waters are well advanced.

A strong regulatory framework will provide the clarity industry needs, and ensure that best available practices are always followed right across the sector and the region. The recent Agreement on ‘Cooperation on Marine Oil Pollution Preparedness and Response in the Arctic’ by the Arctic Council^12^ is a step in the right direction as it aims to strengthen cooperation, coordination and mutual assistance among the Parties on oil pollution preparedness and response in the Arctic.

## Footnotes

1
http://www.miros.no/.

2
http://www.seadarq.com/.

3
http://www.rutter.ca/.

4
http://www.grace-oil-project.eu.

5
http://www.arcticresponsetechnology.org/.

6
http://www.access-eu.org/.

7
http://www.grace-oil-project.eu.

8
https://www.hsva.de/our-facilities/arctic-environmental-test-basin.html.

9
http://www.erdc.usace.army.mil/Locations/CRREL/.

10
http://umanitoba.ca/ceos/research/CMO.html.

11
https://www.sintef.no/en/ocean/laboratories2/.

12
https://oaarchive.arctic-council.org/bitstream/handle/11374/529/EDOCS-2068-v1-ACMMSE08_KIRUNA_2013_agreement_on_oil_pollution_preparedness_and_response_signedAppendices_Original_130510.PDF?sequence=6&isAllowed=y.
